# RNAs as Regulators of Cellular Matchmaking

**DOI:** 10.3389/fmolb.2021.634146

**Published:** 2021-04-09

**Authors:** Nikita Fernandes, J. Ross Buchan

**Affiliations:** Department of Molecular and Cellular Biology, University of Arizona, Tucson, AZ, United States

**Keywords:** RNA scaffolds, RNA decoys, 3′UTR, nascent protein interactions, lncRNA, mRNA

## Abstract

RNA molecules are increasingly being identified as facilitating or impeding the interaction of proteins and nucleic acids, serving as so-called scaffolds or decoys. Long non-coding RNAs have been commonly implicated in such roles, particularly in the regulation of nuclear processes including chromosome topology, regulation of chromatin state and gene transcription, and assembly of nuclear biomolecular condensates such as paraspeckles. Recently, an increased awareness of cytoplasmic RNA scaffolds and decoys has begun to emerge, including the identification of non-coding regions of mRNAs that can also function in a scaffold-like manner to regulate interactions of nascently translated proteins. Collectively, cytoplasmic RNA scaffolds and decoys are now implicated in processes such as mRNA translation, decay, protein localization, protein degradation and assembly of cytoplasmic biomolecular condensates such as P-bodies. Here, we review examples of RNA scaffolds and decoys in both the nucleus and cytoplasm, illustrating common themes, the suitability of RNA to such roles, and future challenges in identifying and better understanding RNA scaffolding and decoy functions.

## Introduction

The cell, with its variety of cellular compartments, varying polarities, competing interactions, and differing sites of molecular synthesis poses challenges to the formation of biomolecular interactions essential to all biological processes. This necessitates ways to bring biomolecules together in a tightly regulated manner. One mechanism for this localization is the use of molecular scaffolds to enable particular interactions while inhibiting off-pathway interactions, thus increasing the efficiencies of the biological processes in which they are involved in Zappulla and Cech (2006). Whereas proteins have long been appreciated to sometimes function as scaffolds, it is becoming increasingly clear that RNA molecules can also facilitate a wide range of interactions among and between proteins and nucleic acids, in many cellular contexts.

In this review, we define an “RNA scaffold” as an RNA molecule capable of bringing together 2 or more macromolecules to form a complex with functional activity. These macromolecules may be proteins, other RNAs or DNA molecules that in the absence of the RNA scaffold do not interact or do so very poorly. By this definition, perhaps the most well-known RNA scaffold molecules are ribosomal RNAs (rRNAs), which provide the structural and catalytic core of ribosomes around which approximately 57–78 ribosomal proteins (depending on species) (Lecompte et al., 2002) assemble to generate functioning ribosomes. Beyond this, long non-coding RNAs (lncRNAs) and even messenger RNAs (mRNAs) are now increasingly being shown to perform a diverse array of scaffolding roles. While rRNAs, bound by ribosomal proteins, are generally regarded as mostly static ribonucleoprotein complexes (RNPs) (Mathis et al., 2017)- although study of “specialized ribosomes” may shift this view (Genuth and Barna, 2018) – lncRNA and mRNAs are generally thought to facilitate more transient, regulatable interactions. Thus, RNAs can scaffold both stable cellular complexes and facilitate transient macromolecular interactions.

RNA can also function as a “decoy” molecule, which we define similarly to a scaffold, except that in this case, two or more macromolecules are brought together by the RNA decoy in a complex that prevents the sequestered macromolecules from forming other interactions and functional complexes at other cellular locations. As with scaffolds, RNA decoy interactions can be transient and regulatable.

RNAs as molecular scaffolds or decoys possess several advantages over other types of biomolecules like proteins and DNA, including: (1) RNAs fold more readily than DNA into complex 3D structures by virtue of diverse secondary and tertiary structural interactions. (2) RNA abundance can be easily and rapidly increased (new transcription) or decreased (RNA decay) while using 1–2 orders of magnitude less energy than occurs for similar regulation of protein (Lynch and Marinov, 2015); indeed translation is the most energy-intensive process in a cell (Topisirovic and Sonenberg, 2011), whereas transcription (barring initiation) is largely an ATP-independent process (Imashimizu et al., 2014). (3) Unlike DNA, RNA is unencumbered with serving as a cell’s nuclear-localized permanent genetic material, thus increasing RNA’s regulatory potential. (4) Compared to proteins, RNA binds other molecules much more efficiently. For instance, 4–17 nts of RNA are capable of binding specific proteins, whereas protein-protein interaction domains typically range from tens to hundreds of amino acids (Prikryl et al., 2011; Chujo et al., 2016; Lunde et al., 2017). Additionally, RNA molecules are generally longer than most proteins (typical size of a globular protein = 5 nm; radius of gyration of mRNA = 16.8–20.8 nm) allowing greater spatial interaction potential with other biomolecules. (5) Evolution of RNA scaffolding functions likely occurs faster and is under less constraint than with protein scaffolds. This is evidenced by lower conservation of lncRNA genes versus protein coding genes (Johnsson et al., 2014), and the more complex protein folding rules and solubility issues that proteins face given their complement of 20 + distinct amino acids.

In this review, we discuss examples of many nuclear and cytoplasmic RNA scaffolds and decoys, some of which regulate relatively stable complexes, while others facilitate transient macromolecular interactions. Concepts of RNA-driven assemblies that have emerged, their physiological importance, and key remaining questions for future study will be highlighted. We will also focus on recent findings of mRNAs acting as cytoplasmic RNA scaffolds, and other means by which nascently translated proteins find their interaction partners. Finally, we discuss steps that can be taken to identify new RNA scaffolds and decoys.

## RNA Scaffolds Can Be Structured or Flexible

RNA scaffolds can generally be classified as either “structured” or “flexible.” RNAs in the former class tend to scaffold stable complexes and are enriched in secondary/tertiary RNA structure, while RNAs in the latter class often aid more transient macromolecular interactions and tend to be less structured.

### RNAs Scaffolding Stable Complexes

In this category, the RNP, composed of the RNA scaffold along with its partner proteins, is sufficiently stable in structure that it can be assessed by X-ray crystallography or by high resolution cryo-electron microscopy (cryo-EM). Zappulla and Cech (Zappulla and Cech, 2006; Zappulla, 2020) have suggested that structured RNPs can further be sub-categorized based on the degree to which the RNA or its protein binding partners drive the overall structure of the RNP.

(i) Structure determined mostly by RNA: In this category, the RNP structure is determined in large part by the folded RNA. An example of this is the ribosome, in which disordered tails or internal loops of proteins constituting the RNP are ordered only after they bind to folded rRNA (Brodersen et al., 2002; Klein et al., 2004). The rRNA structure is so robust that even after degrading most ribosomal proteins (∼95%) from purified ribosomes, rRNA structure and catalytic peptidyl transferase activity still largely persist (Noller et al., 1992). Both sequences and secondary structures of rRNA are highly conserved.
(ii) Structure determined mostly by protein: The RNP has a specific structure that is determined in large part by previously established protein-proteins interactions that constitute the RNP, evident in small nuclear RNPs (snRNPs) and small nucleolar RNPs (snoRNPs). Several snRNP and snoRNP proteins can be crystallized in heteromeric complexes in the absence of their RNA component (Achsel et al., 1998; Deckert et al., 2006; Rashid et al., 2006). That said, during the splicing process, certain snRNPs undergo significant structural re-arrangements dependent on the RNA component; indeed RNA base-pairing driven interactions are essential to spliceosome catalysis (Matera and Wang, 2014; Galej, 2018).

### Flexible RNA Scaffolds Facilitating Transient Macromolecular Interactions

The rest of this review will focus on flexible RNA scaffolds, which nucleate RNPs that lack a rigid structure. In this case, the primary role of the RNA is typically to maintain spatial proximity of macromolecular components, and aid interactions that are often (though not always) more transient in nature. Such RNAs often lack strong sequence conservation and can tolerate large deletions or insertions, but remain functional (Zappulla and Cech, 2006). Conversely, certain structures or subdomains within the flexible RNA scaffold can be of critical functional importance; these can often be transferred in a modular-like fashion to other regions of the RNA and retain functional activity (Stuckenholz et al., 2003; Zappulla et al., 2005; Zappulla and Cech, 2006).

## Nuclear RNA Scaffolds and Decoys

### Introduction to lncRNAs

**FIGURE 1:**
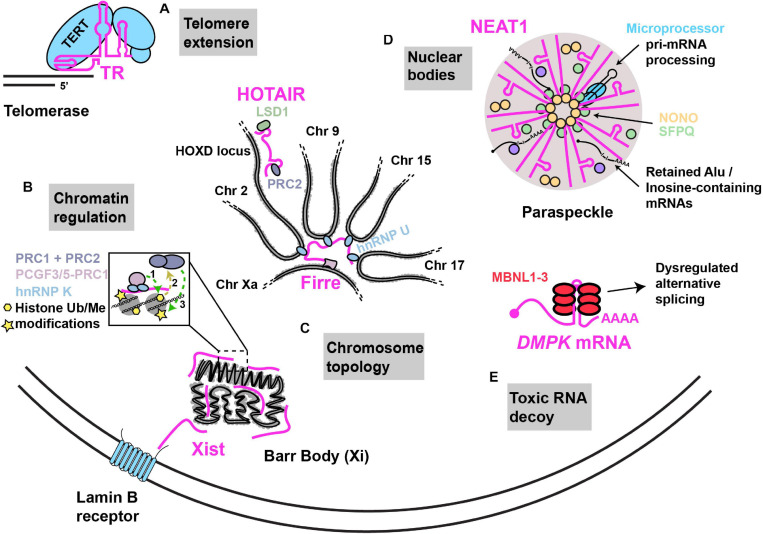
Examples of nuclear RNA scaffolds and decoys. Scaffold and decoy RNAs are depicted in pink. **(A)** Telomere extension: the TR lncRNA scaffolds and provides the telomeric repeat template for the Telomerase complex that consists of the reverse transcriptase protein (TERT) along with other accessory proteins that function in telomere extension. **(B)** Chromatin regulation: *Xist* lncRNA establishes X chromosome inactivation by (1) hnRNP K initiated recruitment of; non-canonical PRC1 complex (Ub ligase) (2), whose activities recruit (3) canonical PRC1 and PRC2 (methyltransferases). HOTAIR recruits PRC2 and LSD1 (demethylase) to modify chromatin at numerous gene loci, including the HOXD locus. **(C)** Chromosome topology: (i) Firre lncRNA expressed from the active X chromosome, establishes chromosome territories by bringing together loci across multiple different chromosomes with the help of interaction with hnRNP U. (ii) *Xist* lncRNA can interact with the Lamin B receptor, resulting in recruitment of the inactivated X chromosome (“Barr body”) to the nuclear lamina. **(D)** Nuclear bodies: NEAT1–2 lncRNA acts as an RNA scaffold driving the assembly of nuclear paraspeckles via its interaction with NONO, SFPQ and several other proteins. NEAT1–2 facilitates NONO-Microprocessor interactions to aid in pri-miRNA processing. Paraspeckles also sequester Inosine-modified RNAs, preventing their export. **(E)** Toxic RNA decoy: CUG repeat expansions in the 3′UTR of DMPK sequester Muscleblind-like (MBNL) proteins, thus impairing alternative splicing.

Most known RNA scaffolds and decoys are defined as lncRNAs, which are a heterogenous group of RNAs > 200 nucleotides in length, and which generally lack long conserved open reading frames, though some may encode short peptides (Slavoff et al., 2013). lncRNAs are transcribed from diverse genomic locations including intergenic regions, and from within protein-coding gene elements including introns, exons, promoters, 5′ and 3′ untranslated regions (UTRs); both sense and antisense lncRNA transcription, with respect to the protein coding gene is observed (Khandelwal et al., 2015). Most lncRNAs are produced by RNA polymerase II, and thus like mRNAs are 5′ methyl-guanosine capped, spliced and 3′ polyadenylated, although the efficiency of these events, particularly splicing, is often lower for lncRNAs (Melé et al., 2017; Mukherjee et al., 2017; Rinn and Chang, 2020). LncRNAs that lack features of mRNA also exist including enhancer-derived ncRNAs (unstable, non-polyadenylated) and other lncRNAs with non-canonical 3′ ends (Zhang Y. et al., 2014; Villegas and Zaphiropoulos, 2015; Li W. et al., 2016). RNA polymerase I, III and, in plants, RNA polymerase V, also generate lncRNAs (Bohmdorfer et al., 2016; Sun et al., 2018). Most lncRNAs localize to the nucleus, possibly due to specific sequence motifs that interact with nuclear RNA binding proteins (Zhang B. et al., 2014; Shukla et al., 2018; Rinn and Chang, 2020), and are typically expressed in low quantities, although their expression ranges from zero to exceeding abundant housekeeping mRNAs (Derrien et al., 2012). LncRNAs tend to exhibit more specific temporal, developmentally regulated and cell-type specific expression patterns than most protein coding genes, suggesting important regulatory functions, as well as tight regulation of lncRNA transcription and decay (Derrien et al., 2012; Gloss and Dinger, 2016). LncRNAs with scaffold or decoy functions impact diverse aspects of nuclear cell biology, select examples of which we discuss below (Figure 1 and Table 1).

**TABLE 1:** Select examples of nuclear and cytoplasmic RNA scaffolds and decoys.

RNA scaffold/decoy	Organism	Type of RNA	Protein interactors	DNA/RNA binding	Guide function	Regulation	Function	Disease relevance	References
**NUCLEAR**									
Alu RNAs	Mammals	mRNA introns, lncRNAs, other RNA molecules	Nucleolin, Nucleophosmin	−	−	−	Formation of 1–3 nucleoli in mammalian cells	−	Caudron-Herger et al., 2016
ANRIL	Mammals	lncRNA	PRC1, PRC2, WDR3, HDAC6	DNA, RNA	−	Transcription, splicing and stability is regulated	(1) Transcription regulation via chromatin modifying complexes; (2) miRNA abundance and activity	Aging, cancers and metabolic diseases	Kong et al., 2018; Zhang et al., 2020
DMPK 3′UTR (CTG repeat expansion)	Mammals	mRNA	MBNL1, MBNL2, and MBNL3	−	−	−	CTG repeat expanded 3′UTR sequesters muscle blind like proteins (toxic)	Myotonic Dystrophy	Miller, 2000
Firre	Mammals	lncRNA	hnRNPU, CTCF	−	−	Firre levels are NF-κB signaling dependent	(1) *In trans*, with hnRNP U, mediates co-localization of multiple chromosomal loci (2) acts *in cis* to maintain XCI by positioning X chromosome near the nucleolus; also preserves H3K27me3	Retinitis Pigmentosa and Periventricular Nodular Heterotopia	Hacisuleyman et al., 2014; Yang et al., 2015; Lu Y. et al., 2017; Balas and Johnson, 2018
HOTAIR	Mammals	lncRNA	PRC2, LSD1-CoREST complex, hnRNPA2/B1, HBXIP, c-Myc, LSD1, HuR	GA rich polypurine DNA motifs on chromatin	Yes	HOTAIR interactions with EZH2 (PRC2 component) regulated by cell-cyle dependent phosphorylation of EZH2	(1) Regulates chromatin dynamics and induces gene silencing, (2) platform for protein ubiquinitation, (3) Scaffolds HBXIP, c-Myc and LSD1 and drives transcription at c-Myc target genes	Multiple cancers	Spitale et al., 2011; Chu et al., 2011; Kaneko et al., 2010; Bhan and Mandal, 2015; Li Y. et al., 2016
Hsr omega	*D. melano gaster*	lncRNA	Nona, Sex-lethal, sans fille, PEP, Hrb87F, Hrp40, Hrb57A, ISWI	−	−	Levels change in response to heat shock	Assembly of Omega speckle in interchromatin space; implicated in hnRNP protein storage, thus affecting mRNA processing/maturation	−	Mallik and Lakhotia, 2009
IGS (Ribosomal Intergenic spacer) RNAs	Mammals	lncRNA	VHL, DNMT1, POLD1, HSP70, MDM2, RPA40, RPA16, NOL1, NOM1, NOP52, PES1, RRP1B, SENP3	−	−	Levels change in reponse to stress	Assembly of Nucleolar detention center/Amyloid bodies; hypothesized cell survival functions by protein sequestration promoting cell dormancy state	−	Audas et al., 2012; Marijan et al., 2019
lncTCF7	Mammals	lncRNA	SWI/SNF complex	DNA	Yes	−	Recruits SWI/SNF complex to TCF7 promoter; drives TCF7 transcription and Wnt signaling activation	Glioma, Hepatocellular Carcinoma	Wang et al., 2015; Gao et al., 2017
MALAT1	Mammals	lncRNA	SRSF1, SRSF2, SRSF3, PC2, Sp1	miRNAs	−	PC2 binding to MALAT1 regulated by growth-signal mediated methylation; methylated state favors TUG1 binding (see below).	(1) Debated role in regulating phosphorylation and expression pattern of SR proteins, and thus alternative splicing; (2) PC2 methylation status regulates localization of growth-control genes between polycomb bodies (transcriptionally silent) and interchromatin granules (transcriptionally active). MALAT1 scaffolds the latter and facilitates E2F1 SUMOylation, activating growth-control gene program (3) Activation of LTBP3 transcription via Sp1 recruitment to promoter	Multiple cancers	Tripathi et al., 2010; Yang et al., 2011; Zhang et al., 2012; Li B. et al., 2014
MANTIS	Mammals	lncRNA	BRG1 (SWI/SNF complex)	−	−	Downregulated in lungs in idopathic pulmonary arterial hypertension patients	Upregulation of endothelial genes by promoting BRG1-BAF155 interaction and BRG1 ATPase activity	Hypertension	Leisegang et al., 2017
Mei RNA	*S. pombe*	lncRNA	Mei2, Mmi1	−	−	Transcription pregulated by starvation by Ste11	Assembly of Mei2 dot, regulation of meiotic gene expression, induction of homologous chromosome pairing	−	Yamashita et al., 1998; Yamashita, 2019
Myheart	Mammals	lncRNA	BRG1 (SWI/SNF complex)	−	−	Transcription inhibited by BRG1-HDAC-PARP chromatin repressor complex in heart tissue by pathological stress	Binds to BRG1 sequestering it, protects heart from hypertrophy	Cardiac hypertrophy	Han et al., 2014
NEAT1–2, NEAT1–2	Mammals	lncRNA	PSPC1, SFPQ, NONO, RBM14, HNRNPK, FUS, DAZAP1, HNRNPH3, HNRNPA1, HNRNPR, HNRNPUL1, TDP-43, BRG1, BRM, BAF155	RNA	−	Levels change in response to stress, viral infection and development	Assembly of paraspeckles, which are implicated in transcription, splicing RNA processing and export regulation	−	Clemson et al., 2009
Oct4P4	Mammals	lncRNA	HP1a	DNA	Yes	−	Facilitates transcriptional silencing of Oct4 gene in differenitiating mouse embryonic stem cells	−	Scarola et al., 2015
p21	Mammals	lncRNA	hnRNP K	DNA	Yes	p53 activity (e.g., following DNA damage) induces transcription	RNP imparts specificity to genes repressed by p53 induction	−	Huarte et al., 2010
PARTICLE	Mammals	lncRNA	G9a, SUZ12	DNA	Yes	−	Forms DNA-RNA triplex at MAT2A locus. Recruits transcription-repressive complexes (G9a lysine methyltransferase, PRC2) resulting in methylation and MAT2A repression	−	O’Leary et al., 2017
PINCR	Mammals	lncRNA	Matrin3, p53	−	−	p53 activity (e.g., following DNA damage) induces transcription	Upregulation of genes involved in cell cycle arrest and apoptosis	Colorectal cancer	Chaudhary et al., 2017
Satellite III	Mammals	lncRNA	SRSF1, SAFB, TDP-43, HSF1, BRG1, NFAT5	−	−	Levels change in response to heat shock	Assembly of Nuclear stress body	−	Valgardsdottir et al., 2005
THRIL	Mammals	lncRNA	hnRNP L	DNA	Yes	−	Facilitates transcription of several immune response genes, including TNFα in macrophages	−	Li Z. et al., 2014
TR	Eukaryotes	lncRNA	TERT, Dyskerin complex, TCAB1	DNA	−	Deregulation in cancer cells; TERC maturation regulated	(1) Telomerase-mediated telomere extension. (2) Transcriptional regulation of certain genes	Multiple cancers	Collins, 2008; Liu et al., 2019; Shay and Wright, 2019
TUG1	Mammals	lncRNA	Methylated PC2 in Polycomb bodies (PcGs)	−	−	see MALAT1 details (above)	TUG1 scaffolds Polycomb bodies; binds methylated PC2 and various transcriptional repressor complexes	−	Yang et al., 2011
Xist	Mammals	lncRNA	PRC1, PRC2, LBR, hnRNP U, hnRNP K, SHARP, SMRTY, HDAC3	DNA	−	Xist levels change through embryonic development	X-chromosome inactivation (XCI) by *cis-*recruitment of numerous chromatin modifiers; also induces Xi lnuclear membrane ocalization via Lamin B receptor interactions	−	Cerase et al., 2015; Lu Z. et al., 2017
Y3** RNA	Mammals	Y RNA	F	RNA	Yes	−	3′ end processing of histone pre-mRNAs	−	Köhn et al., 2015
**Cytoplasmic**									
1/2-sbsRNAs	Mammals	lncRNA	Staufen, Upf1	mRNA	Yes	−	Alu element-containg lncRNAs bind 3′UTR sites in various mRNAs; resulting duplexes recruit Staufen, Upf1 and elict Staufen-mediated decay	−	Gong and Maquat, 2011
7SL	Eukaryotes	lncRNA	SRP14, SPR9, SRP19, SRP54, SRP68, SRP72	−	−	Levels downregulated by mir-125b in Zebrafish embryos	(1) Scaffolds signal recognition particle (SRP); recognized N-terminal signal peptides for secretory/membrane protiens, stalls translation and aids translocation of nascent peptides into ER. (2) Regulates p53 translation via competition with HuR for p53 3′UTR binding	−	Rosenblad et al., 2009; Jalali et al., 2013; Abdelmohsen et al., 2014
BIRC3 3′UTR	Mammals	mRNA	HuR, Staufen, IQGAP1, RALA	−	−	Alternative cleavage and polyadenylation generates short and long 3′UTR isoforms	Long 3′UTR BIRC3 mRNA binds HuR and Staufen; leads to formation of various specific BIRC3 protein complexes implicated in chronic lymphocytic leukemia pathology	Chronic Lymphocytic Leukemia	Lee and Mayr, 2019
CD47 3′UTR	Mammals	mRNA	HuR, SET, RAC1	−	−	Alternative cleavage and polyadenylation generates short and long 3′UTR isoforms	Long 3′UTR CD47 mRNA binds HuR and SET; interacts with nascent CD47 and promotes RAC1 interaction; leads to CD47 translocation to plasma membrane for anti-phagocytic function	−	Berkovits and Mayr, 2015; Ma and Mayr, 2018
HOTAIR	Mammals	lncRNA	HuR	miRNAs	−	Levels regulated by HuR binding and subsequent recruitment of let-7 miRNA/RISC complex, which degrades HOTAIR. Accumulates with low HuR abundnace during senesence	(1) Scaffolds two ubiquitin ligases DZIP3 and MEX3B and their substrates Snurportin 1 (SNUPN) and Ataxin-1 (ATXN1), respectively; promotes SNUPN and ATXN1 ubiquitination and degradation. (2) Acts as miRNA decoy	Multiple cancers	Yoon et al., 2013; Bhan and Mandal, 2015
MALAT1	Mammals	lncRNA	−	miRNAs	−	See details on nuclear MALAT1	miRNA decoy function – counteracts suppression of oncogenes	Multiple cancers	Salmena et al., 2011; Luan et al., 2016; Wang et al., 2016
p21	Mammals	lncRNA	HuR, RNA-induced silencing complex (RISC), HIF1A, von Hippel-Lindau (VHL) protein	mRNA, miRNA (let-7)	−	Levels downregualted by HuR binding and subsequent recruitment of let-7 miRNA/RISC complex. Accumulates with HuR depletion and hypoxia	(1) p21 can form partial hybrids with target mRNAs like CTNNB1 and JUNB mRNAs leading to recruitment (via unclear mechanism) of translational suppressors RCK/p54 and FMRP to mRNAs. (2) Upon hypoxia, accumulated p21 binds to HIF1A and the von Hippel-Lindau protein. This allows HIF1A accumulation as VHL-mediated HIF1A ubiquitination is prevented, in turn promoting glycolysis	Multiple cancers	Yoon et al., 2012; Yang et al., 2014
RPS28B 3′UTR	*S. cerevisiae*	mRNA	Edc3	−	−	−	*cis*-translation- mRNA 3′UTR recruits Edc3 that enables its interaction with Rps28 protein translated off of the mRNA	−	Fernandes and Buchan, 2020
Y3 precursor RNA	Mammals	Y RNA	HuD	−	−	−	Inhibits function of HuD which regulates alternative splicing and polyadenylation, localization, translation and stability of neuronal mRNAs.	−	Bronicki and Jasmin, 2013

*Additional details on discussed RNA scaffolds and decoys, as well as other non-discussed RNA scaffolds and decoys of note.*

### TR lncRNA: Template and Scaffold of Telomerase

An elegant example of an RNP featuring a flexible RNA scaffold is the telomerase enzyme that functions in the maintenance of genome stability by counteracting the loss of DNA at chromosomal ends following rounds of DNA replication (Figure 1). Telomerase is scaffolded by the telomerase RNA (TR; also named telomerase RNA component; TERC), a lncRNA that functions both as a template in the reverse transcriptase-driven extension and maintenance of telomeric DNA, and which binds and spatially organizes various telomerase complex proteins including the telomerase reverse transcriptase (TERT), and several other species-specific accessory proteins (Chan and Blackburn, 2004; Wu et al., 2017). RNA structural modeling in different species suggests that TR usually harbors 3 long arms that each bind to specific proteins of the telomerase complex (Zappulla and Cech, 2006; Zhang et al., 2011; Zappulla, 2020). Each arm consists of double-helical regions with several internal loops and bulges, features that provide flexibility to RNAs in solution (Nowakowski and Tinoco, 1997). Another conserved feature of TR is the presence of a template/pseudoknot domain (“t/PK”) which contains the template used for replicating telomeric sequence, and a pseudoknot implicated in aiding TERT catalysis, template positioning and telomerase assembly (Chen and Greider, 2005; Theimer et al., 2005; Qiao and Cech, 2008; Wang et al., 2016). TR length ranges significantly between species from 150nts in ciliates to 3kb in certain yeast species. Furthermore, large stretches of TR can be deleted or domains transferred to other parts of TR without significant loss of function suggesting that TR structural flexibility allows maintenance of function (Livengood et al., 2002; Zappulla and Cech, 2006).

Telomerase complex assembly, and particularly recruitment to telomeres is often transient, and typically regulated in a cell-cycle dependent manner (Vasianovich and Wellinger, 2017). In yeast, assembly of the active telomerase enzyme depends on binding of a particular subunit (Est3) to the reverse transcriptase, which in turn must already be bound (along with another subunit, Est1) to the TR. Such assembly only occurs in G2/M, and while protein interaction domains of telomerase subunits are known, the underlying regulation of this process remains unclear (Tucey and Lundblad, 2014). Interestingly, in humans, TR and TERT remain stably associated throughout the cell cycle based on competitor interaction studies, but a key telomerase subunit (Telomerase Cajal body protein 1; TCAB1) decreases interaction with the TR during M phase. Finally, single-molecule imaging of TERT in mammalian cells revealed that during S-phase, telomerase mostly exists in a freely diffusing nuclear state, and only transiently interacts with telomeric ends (Schmidt et al., 2016). Specifically, TERT exhibited ∼2400 telomere encounters in an 8 hr S-phase, with only rare long-lasting telomerase-telomere interactions of ∼3.7 mins observed in about 4% of cells.

### lncRNAs Regulating Chromosome Topology and Nuclear Localization

Many lncRNAs associate with chromatin, and can enact either widespread or local changes in chromatin conformation that can regulate chromosome topology, localization and the process of transcription (Werner and Ruthenburg, 2015).

The most studied lncRNA modifier of chromosome topology is *Xist* (X-inactive specific transcript), a 17 kb lncRNA that mediates X chromosome inactivation (XCI), a method of sex-chromosome gene dosage compensation where one X chromosome in every female cell is almost completely transcriptionally silenced at random (Chow et al., 2005). Following transcription, *Xist* lncRNA coats and is tethered to the entire future inactivated X chromosome (“Xi”) by an AT-rich DNA interacting protein called hnRNP U (Hasegawa et al., 2010). Ultimately, *Xist* promotes condensation of the Xi into a compact structure termed the Barr body, which is repositioned either to the nuclear lamina, or to peri nucleolar regions (Andrulis et al., 1998; Zhang et al., 2007; Rego et al., 2008); in both cases, these regions are enriched in and likely help maintain transcriptionally inactive heterochromatin (Clemson et al., 1996; Padeken and Heun, 2014). *Xist* harbors several conserved, structured tandem-repeat regions, named A to F, that are implicated in *Xist* association with the Xi, *Xist* spreading across Xi, and *Xist* association with transcriptional silencing complexes (Wutz et al., 2002; Pintacuda et al., 2017b). Although many *Xist* structural domains and protein interactions have been described (Chu et al., 2015; Smola et al., 2016), one intriguing example is interaction of a region of *Xist* overlapping it’s 5′ localized F-repeat with the Lamin B receptor (LBR), a *trans-*membrane protein embedded in the inner nuclear membrane (Chen et al., 2016). This interaction induces Xi localization to the nuclear lamina, which may facilitate *Xist* spreading on Xi and transcriptional silencing due to chromosome immobilization (Chen et al., 2016; Figure 1).

Another repeat-containing lncRNA that affects chromosome topology is Firre, which unlike *Xist*, appears to be transcribed predominantly from the active X chromosome (“Xa”) (Fang et al., 2020) and can associate *in trans* with a region of the Xi. Firre expression also promotes Xi positioning at the nuclear lamina and near the nucleolus in a manner dependent on the zinc finger transcription factor CTCF (Deng et al., 2015; Fang et al., 2020). Firre also helps preserve H3K27me3, a repressive chromatin mark, via Polycomb repressive complex 2 targeting (PRC2) to Xi (Yang et al., 2015).

Firre also regulates inter-autosomal interactions by interacting via its repeat domains with hnRNP U (Figure 1). This drives formation of nuclear domains in proximity to Firre’s genomic locus on the Xa, which induces co-localization of loci on chromosomes 2, 9, 15, and 17 (Hacisuleyman et al., 2014). Both deletion of the Firre locus and hnRNP U knockdown result in loss of colocalization of the *trans-*chromosomal interacting loci (Hacisuleyman et al., 2014). Firre null cells also exhibit > 1000 genes with significant changes in gene expression as assessed by RNA-seq (Hacisuleyman et al., 2014), while Firre KO mice exhibit close to 4000 differentially expressed genes across multiple tissues, and exhibit a specific defect in hematopoiesis (Lewandowski et al., 2019). Thus, Firre appears to act as a scaffold that initiates and organizes specific chromosomal domains within the nucleus through specific chromatin interactions, which in turn regulates diverse gene expression programs (Nakagawa and Hirano, 2014).

### lncRNAs Regulating Localized Chromatin Modification and Transcription

LncRNAs can regulate transcriptional activity either by regulating chromatin modification and thus conformation, or by directly affecting transcription. This is achieved by lncRNAs recruiting factors such as histone modifying complexes, DNA methyltransferases, transcription factors and other DNA-binding transcriptional regulators to specific genomic loci. This recruitment process may involve a lncRNA binding to a specific loci, or an entire chromosome (as discussed for *Xist*), in *cis* or in *trans*, by base pairing with other RNAs, by the lncRNA binding directly to DNA, or by RNA-protein interactions (Long et al., 2017).

Numerous *Xist*-interacting proteins have been identified by various cross linking and mass-spectrometry approaches (McHugh et al., 2015; Almeida et al., 2017; Brockdorff, 2017; Pintacuda et al., 2017a), many of which are chromatin modifiers, which likely help initiate and maintain XCI. In one study, amongst other *Xist* directly interacting proteins including hnRNP U and LBR, a transcriptional repressor named SHARP (SMRT and HDAC associated repressor protein) was identified as an *Xist* binder which in turn recruits SMRT (silencing mediator of retinoid and thyroid receptor) and histone deacetylase 3 (HDAC3) (Chu et al., 2015; McHugh et al., 2015). More recent work has identified a 600 nt region of the *Xist* RNA overlapping the B repeat, termed the *Xist* RNA Polycomb interaction domain (XR-PID), which is bound by the RNA-binding protein (RBP) hnRNP K (Pintacuda et al., 2017a). This promotes recruitment of a non-canonical Polycomb chromatin-modifying complex (PCGF3/5-PRC1) which ubiquitinates Histone H2A. This serves as a recruitment signal for other PRC1 complexes and the PRC2 Polycomb complex, which promotes H3K27me3 repressive modification (Almeida et al., 2017), a feature of XCI (Wutz, 2011; Figure 1). Interestingly, PRC2 Xi recruitment is inhibited in SHARP or HDAC3 knockdown cells (McHugh et al., 2015), though understanding of how histone deacetylation and ubiquitination co-operate in PRC1 and 2 recruitment, in an *Xist* dependent manner, remains unclear. In summary, *Xist* harbors a complex, *cis*-acting RNA-scaffolding function, involving interactions with several chromatin modifiers and DNA binding proteins.

RNA scaffolds can also drive recruitment of chromatin modifiers *in trans* at specific gene loci; this was first reported for the 2.2 kb human HOX antisense intergenic RNA (HOTAIR), which is encoded on chromosome 12 at the HOXC locus (Rinn et al., 2007). HOTAIR appears capable of simultaneously binding PRC2 via a 5′ region and Lysine-specific demethylase 1 (LSD1) via a 3′ region (Tsai et al., 2010; Figure1). Chromatin-IP analysis (ChIP) revealed that depletion of HOTAIR results in striking transcriptional activation of a 40 kb region of HOXD locus on chromosome 2 owing to loss of PRC2-mediated H3K27me3 and gain of LSD1-regulated H3K4me2. HOTAIR depletion also alters these chromatin modifications, and PRC2 and/or LSD1 binding, at hundreds of other genes (Tsai et al., 2010). Besides implications for a role in development, given the nature of HOX gene function in embryogenesis (although this is controversial (Selleri et al., 2016)) HOTAIR is also overexpressed and may affect pathology in various cancers (Hajjari and Salavaty, 2015).

LncRNAs can also directly affect transcription by interacting with transcription factors and the transcriptional apparatus. For instance, in breast cancer cells, HOTAIR scaffolds a complex involving LSD1 (Amente et al., 2010a, b) and HBXIP, which is a co-activator and binding partner of the oncogenic transcription factor c-Myc. Ultimately, c-Myc recruitment of the HBXIP/HOTAIR/LSD complex transcriptionally activates c-Myc target genes which in turn drives c-Myc-mediated oncogenesis (Li Y. et al., 2016). Many other lncRNAs interact with chromatin modifying proteins to regulate chromatin modification, or interact with transcription factors, resulting in either transcriptional activation or repression. We guide readers to these excellent reviews (Rinn and Chang, 2012; Rutenberg-Schoenberg et al., 2016; Kopp and Mendell, 2018).

### RNAs Scaffolding Nuclear Bodies: Paraspeckles, Toxic RNA Foci, and Nucleoli

lncRNAs can function as RNA scaffolds for nuclear bodies (Chujo et al., 2016), which are membrane-less liquid-like organelles rich in protein and RNAs. These include nucleoli, paraspeckles, Cajal bodies and Polycomb bodies (Mao et al., 2011). Very generally, nuclear bodies can act as sites of biogenesis, maturation, storage, and sequestration of specific RNPs or regulators of nuclear RNA processes. Some nuclear bodies harbor specific lncRNAs implicated in their scaffolding which tend to be characterized by repetitive sequences. These may either concentrate biomolecules in a nuclear body, and thus facilitate a molecular process, or sequester in a seemingly inert state specific biomolecules away from other cellular locations, often altering gene expression mechanisms in the process. Thus, nuclear body RNA scaffolds can also sometimes be considered as decoys.

Nuclear paraspeckles, which are implicated in regulating transcription, splicing, RNA processing and export, are scaffolded by the nuclear-enriched abundant transcript 1 (NEAT1) lncRNA (Bond and Fox, 2009; Figure 1). Paraspeckles are nucleated at NEAT1 loci, particularly following transcription of the NEAT1_2 (22.7 kb) lncRNA isoform; a second isoform, NEAT1_1 (3.7 kb) has a more supplementary role in paraspeckle formation (Clemson et al., 2009; Naganuma et al., 2012). NEAT1 is essential for the formation and maintenance of paraspeckles based on knockdown and over-expression studies. Several RBPs localize in paraspeckles (Naganuma et al., 2012; Kawaguchi et al., 2015), including NONO, PSPC1, and SFPQ; all 3 of these are part of the Drosophila Behavior/Human Splicing family of proteins and function in numerous steps of nuclear mRNA metabolism including transcription, splicing, processing and export (Knott et al., 2016). Careful truncation analyses, RBP-NEAT1 tethering assays and microscopy have identified that the middle domain of NEAT1–2 contains functionally redundant repetitive sequences that bind NONO and SFPQ. A dimerization domain of NONO also drives paraspeckle assembly, a process that exhibits phase transition-like mechanisms similar to other RNP membrane less bodies (Boeynaems et al., 2018; Yamazaki et al., 2018). Consistent with the importance of the central NEAT1_2 region, electron and super-resolution microscopy indicates that the middle region of NEAT1–2 locates to the inner core of paraspeckles, whereas 5′ and 3′ ends of NEAT1_2 are located in the outer shell (Souquere and Pierron, 2015; West et al., 2016).

NEAT1 and paraspeckles regulate gene expression in diverse ways through both scaffolding and decoy functions. As a scaffolding example, NONO-SFPQ heterodimeric complexes bind to and enhance processing of select pri-miRNAs by the Microprocessor complex, which conducts the first step in miRNA processing (Jiang et al., 2017). NEAT1 binds both NONO-SFPQ dimers (presumably in the central region) and the Microprocessor complex at a 3′ stem loop motif (also implicated in regulating NEAT1_2 stability (Yamazaki et al., 2018)). Importantly, knockdown or knockout of NEAT1 strongly impedes NONO interactions with Microprocessor, and impairs pri-miRNA processing, indicating NEAT1 scaffolds Microprocessor interactions with NONO-SFPQ to aid the production of mature miRNA (Jiang et al., 2017; Figure 1).

A decoy function of paraspeckles and NEAT1 is sequestration, and thus limiting export of mRNAs harboring inverted repeat Alu elements in their 3′ untranslated region (UTR; Alu elements are transposable elements that account for 11% of the human genome, and which likely derive from 7SL ncRNA (Deininger, 2011); discussed later). This feature makes RNAs targets for Adenosine-to Inosine (A to I) editing by Adenosine deaminase enzymes (Athanasiadis et al., 2004). Paraspeckles are enriched in A to I hyper-edited RNA (Zhang and Carmichael, 2001), owing to such modified RNA being bound by NONO, and several such mRNAs are retained in the nucleus (or specifically, where examined microscopically, in paraspeckles themselves). This depends on the mRNAs harboring a modified Alu element in their 3′UTR, as well as the presence of NEAT1 expression and paraspeckle formation (Prasanth et al., 2005; Chen et al., 2008; Chen and Carmichael, 2009; Osenberg et al., 2009). Such mRNA sequestration can be regulated; for example, mCAT2 mRNA is released from paraspeckles following a stress-activated 3′UTR cleavage event (Prasanth et al., 2005) and may in turn regulate innate immune and inflammatory responses (Lowenstein and Padalko, 2004; Pisani and Baron, 2019).

Another decoy function of NEAT1 is sequestration of transcription regulating factors. In response to either proteasomal inhibition or viral infection, NEAT1 RNA abundance increases > 4 fold, causing significant paraspeckle enlargement and sequestration of SFPQ. This titrates SFPQ away from specific promoters, altering mRNA expression in a context dependent manner (Hirose et al., 2014; Imamura et al., 2014). In NEAT1 knockdown or knockout contexts, failure to transcriptionally regulate these mRNAs correctly following proteasomal inhibition or viral infection leads to increased cell death and impaired innate immune responses, respectively (Hirose et al., 2014; Imamura et al., 2014). Thus, regulation of NEAT1 expression and paraspeckle formation may play a key role in mediating stress-inducible gene expression responses by sequestration of both mRNA and proteins.

While repetitive sequences often are of functional benefit to lncRNA scaffolds (Duszczyk et al., 2011; Yamazaki et al., 2018), aberrant repeat expansion in mRNAs is implicated in playing decoy functions that contribute to various neurodegenerative diseases. To date, 10 neurodegenerative diseases including Myotonic dystrophy type 1 and 2, Fragile X-associated tremor ataxia syndrome, Amyotrophic Lateral Sclerosis (ALS), Huntington’s disease-like 2 and a variety of other Ataxia conditions feature repetitive sequence expansions in regions including UTRs, introns, promoter and variant exon sequences (Wojciechowska and Krzyzosiak, 2011; Swinnen et al., 2020). Myotonic dystrophy type 1, caused by expansion of a CTG repeat in the 3′UTR of the *DMPK* gene represents the clearest example of an RNA decoy disease (Mastroyiannopoulos et al., 2010; Figure 1). This expansion results in the formation of a stable hairpin conformation that leads to accumulation of RNA nuclear foci (Taneja et al., 1995; Davis et al., 1997; Tian et al., 2000); why such RNAs fail to export is still unclear (Smith et al., 2007; Mastroyiannopoulos et al., 2010). In turn, these RNA foci sequester specific RBPs, particularly muscleblind-like (MBNL) family proteins MBNL1, MBNL2, and MBNL3 (Miller, 2000; Mankodi et al., 2001). These proteins regulate alternative splicing in a tissue-specific manner, and splicing defects are observed in various mouse models and patients (Swinnen et al., 2020). Other means of repeat expansion toxicity, including repeat-associated translation of repeat sequences via a non-canonical translation mechanism, and loss of protein expression (owing to poor transcription or enhanced mRNA decay) are also proposed mechanisms in many of the above-mentioned diseases (Swinnen et al., 2020).

Finally, the nucleolus is also scaffolded by at least two distinct RNA molecules. In humans, nucleoli form due to juxtaposition of rDNA arrays, located on five chromosomes in strongly euchromatic regions, to typically form 1–3 nucleoli per cell (Savino et al., 2001). The essential rRNA transcription factor UBF1 binds to and maintains rDNA arrays in an open chromatin state which initiates nucleoli formation (Grob et al., 2014). This involves rRNA transcription itself, which confers spatiotemporal regulation on nucleoli formation, whose assembly varies during embryogenesis and cell cycle progression (Hernandez-verdun, 2011; Berry et al., 2015; Falahati et al., 2016). However, a long-stranding puzzle in the field was that RNAP II inhibition strongly dispersed nucleoli into multiple small foci, even though RNAP II activity is negligible in nucleoli (Carmo-Fonseca, 2015). An answer to this conundrum came from sequencing of nucleolar RNA fractions, and subsequent RNA fluorescence *in situ* (FISH) experiments, which identified a strong enrichment of Alu repeat-containing RNAs (AluRNAs) in nucleoli that mostly derive from introns and subsequent pre-mRNA splicing of multiple genes (Caudron-Herger et al., 2015). Knockdown and ectopic expression of AluRNAs confirmed a clear role in stimulating assembly of normal-sized nucleoli, which involves a scaffolding function whereby AluRNAs bind nucleolin and nucleophosmin, two abundant structural proteins of the nucleolus that harbor low-complexity domains (possibly aiding phase-transition like properties of the nucleolus), and which also bind UBF1 (Li et al., 1996; Hisaoka et al., 2010). Thus, *trans* acting AluRNA scaffolds appear to “glue” small nucleolar bodies together (Caudron-Herger et al., 2015, 2016). Critically, depletion of AluRNAs or impairment of RNAP II transcription strongly reduces rRNA transcription, whereas AluRNA overexpression enhanced rRNA transcription, arguing that AluRNA scaffolding facilitates nucleolar functions in rRNA production (Caudron-Herger et al., 2015, 2016). Whether other nucleolar functions (Iarovaia et al., 2019), including roles in telomerase (Fu and Collins, 2007) and signal recognition particle assembly (Massenet, 2019) (see below) are impacted by AluRNA-driven scaffolding is unknown but worthy of future study.

## Cytoplasmic RNA Scaffolds

**FIGURE 2:**
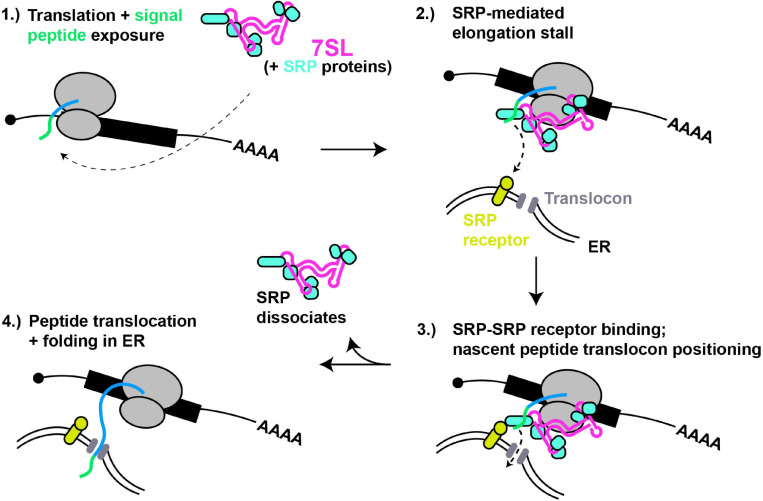
7SL – a paradigm of cytoplasmic RNA scaffolding. **(1)** Translation of proteins with N-terminal signal peptides (green) are bound by SRP complex, scaffolded by 7SL. **(2)** Signal peptide binding induces SRP conformational change and tighter binding, thus block ribosomal A-site and stalling translation elongation. **(3)** SRP interaction with SRP receptor positions nascent peptide for entry into ER translocon. **(4)** Dissociation of SRP relieves elongation stall, and nascent peptide extends into and folds within ER lumen.

Several examples of flexible cytoplasmic RNA scaffolds and decoys have recently come to light, particularly involving cytoplasmic lncRNAs and mRNAs. These, together with a long-known ribosome-associated flexible RNA scaffold are now discussed (Figure 2 and Table 1).

### 7SL Scaffolds Signal Recognition Particle

The signal recognition particle (SRP) is a well-studied RNP complex that is scaffolded by an RNA Polymerase III-derived RNA (7SL; Figure 2). SRP recognizes and binds to N-terminal signal peptides present on proteins destined for secretion or membrane localization as they emerge from a ribosome. SRP binding to signal peptides causes a stall in translation elongation, and targets the ribosome to a translocon pore in the ER membrane where translation then resumes, resulting in the unstructured nascent peptide being threaded into the ER lumen, where folding and later trafficking can ultimately occur (Keenan et al., 2001).

The human 7SL lncRNA is a ∼300 nt long structured RNA characterized by helical regions forming 2 domains separated by a flexible linker, a smaller domain (Alu) and a larger domain (S), with each part recruiting specific proteins (6 in total) that form the SRP (Pool, 2005). The Alu domain of 7SL preferentially binds the SRP14/SRP9 heterodimer whose function involves binding the ribosome and inhibiting translation elongation after signal recognition by obscuring access to the ribosomal A-site, while the S domain binds the heterodimer SRP68/SRP72, SRP19 and SRP54, and binds in proximity to the nascent peptide tunnel (Siegel and Walter, 1988; Wild et al., 2001). Besides signal peptide binding (via SRP54), the S domain also mediates SRP interactions with the SRP receptor at the ER membrane (Gowda et al., 1997; Pool et al., 2002). Crystal structure analyses suggests the SRP68/SRP72 heterodimer binding of 7SL drives an initial conformation change enabling proper interaction of SRP with the ribosome (Grotwinkel et al., 2014). Furthermore, Cryo-EM studies indicate that SRP bound to the elongating ribosome exists in at least two distinct states. In the “scanning” state, SRP samples nascent peptides for signal peptide sequences. If none are detected, elongation factor binding readily displaces the Alu domain away from the ribosomal A-site, whereas in the “engaged state,” with SRP54 bound to the nascent peptide, the Alu domain remains robustly bound near the A-site, thus inhibiting translation (Voorhees and Hegde, 2015). Thus, dynamic flexibility of the 7SL RNA scaffold is critical to SRP function.

### Cytoplasmic lncRNAs as RNA Scaffolds and Decoys

While most lncRNAs exhibit a significant bias toward nuclear localization, many also localize in the cytoplasm and thus unsurprisingly, are increasingly being linked to an array of diverse scaffolding and decoy roles.

**FIGURE 3:**
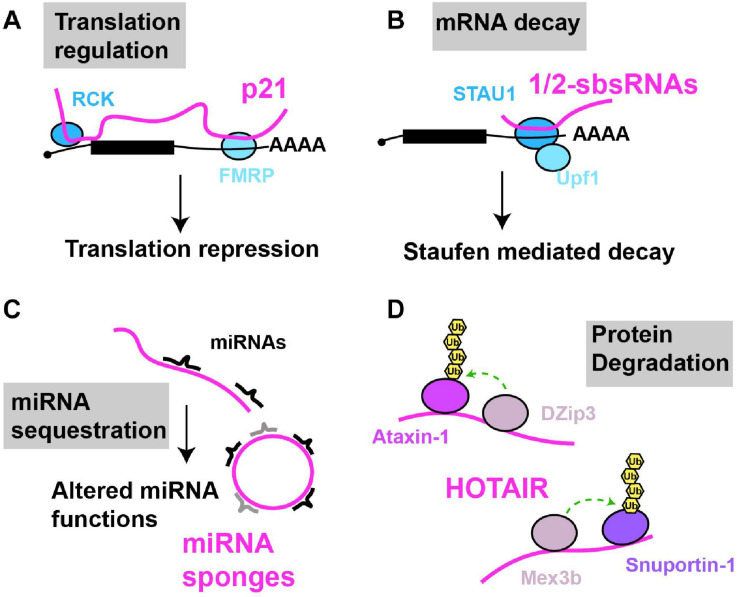
Examples of cytoplasmic RNA scaffolds and decoys. Scaffold and decoy RNAs are depicted in pink **(A)** mRNA translation: lncRNA-p21 partially base pairs with target mRNAs leading to the recruitment of translation repression factors like RCK and FMRP, thus inhibiting mRNA translation. **(B)** mRNA decay: 1/2sbsRNAs base pair with target mRNAs; the resulting dsRNA recruits Staufen, leading to staufen-mediated mRNA decay. **(C)** miRNA sequestration: various lncRNAs (linear and circular) in multiple species, regulated in a tissue, developmental or environmentally sensitive manner, can base-pair with and sequester miRNAs, preventing their regulation of mRNA translation or decay. **(D)** Protein degradation: HOTAIR lncRNA binds to two ubiquitin ligases and their substrates causing their ubiquitination and degradation.

lncRNAs can bind to mRNA *in trans*, and scaffold complexes that impact mRNA function. For example, numerous lncRNAs harboring Alu elements (“1/2-sbsRNAs”) were identified that form partially complementary duplexes *in trans* with Alu elements present in various mRNA 3′UTRs (Gong and Maquat, 2011; Figure 3). Upon lncRNA-mRNA binding, the resulting double stranded RNA serves as a binding site for the RBP Staufen, which in turn recruits the RNA helicase Upf1, resulting in degradation of many target mRNAs by a process termed Staufen mediated decay (SMD) (Kim et al., 2005). SMD regulation by lncRNAs is complex, given that a) several different Alu-containing lncRNAs can promote decay of the same mRNA; b) a given Alu-containing lncRNA can regulate several mRNAs; and c) not all mRNA targets with complementarity to a given Alu-containing lncRNA necessarily undergo decay, possibly owing to other structural aspects of the mRNA-protein complex (mRNP) (Gong and Maquat, 2011; Gong et al., 2012; Park and Maquat, 2013). In another case, p21, a highly studied lncRNA transcriptional target of p53 with many nuclear functions, also forms hybrids with target cytoplasmic mRNAs (Figure 3). Specifically, p21 exhibits partial base pair complementarity and affinity purifies with CTNNB1 and JUNB mRNAs, which encode oncogenic proteins β-catenin and JunB (Yoon et al., 2012). Based on knockdown studies, p21 exerts an inhibitory effect on translation of these mRNAs, rather than mRNA decay. Consistent with this, p21 also affinity purifies with the translational repressors RCK and FMRP, and fractionates in polysomes, suggesting that p21 pairs to and recruits translation repressors to mRNA targets (Yoon et al., 2012).

A second example of 3′UTR co-translationally scaffolding assembly of protein complexes involves the Baculoviral IAP repeat-containing protein 3 (BIRC3) gene. BIRC3 encodes a ubiquitin ligase, and alternative cleavage and polyadenylation generates short (BIRC3-SU) and long (BIRC3-LU) 3′UTR mRNA isoforms, the latter of which is significantly upregulated in chronic lymphocytic leukemia (CLL) cells (Lee and Mayr, 2019). While not affecting BIRC3 mRNA or protein localization, or protein levels, BIRC3-LU is specifically bound by HuR and Staufen, which in turn recruits trafficking-regulating proteins (IQ motif containing GTPase IQGAP1, and the Ras GTPase RALA) (Lee and Mayr, 2019). These interact with nascently translated BIRC3 such that the 3′UTR scaffolds formation of a BIRC3-RALA-IQGAP1 complex. This complex in turn binds and promotes plasma membrane recycling of a receptor protein called CXCR-4, which promotes CLL progression by aiding malignant B cell migration and survival via targeting to protective bone marrow niches (Burger et al., 2005). Finally, the BIRC3-LU 3′UTR, distinct from the BIRC3-SU isoform, uniquely binds proteins involved in mitochondrial biology and chromatin remodeling, suggesting that multiple functionally distinct protein complexes may be scaffolded by this particular 3′UTR (Lee and Mayr, 2019).

While best known for its nuclear function HOTAIR is also found in the cytoplasm, particularly under cellular conditions such as senescence (Yoon et al., 2013). Cytoplasmic HOTAIR serves as a binding site for two ubiquitin ligases DZIP3 and MEX3B, and their substrates Snurportin 1 (SNUPN) and Ataxin-1 (ATXN1); this binding leads to increased SNUPN and ATXN1 ubiquitination and their degradation. HOTAIR accumulation is necessary for eliciting proper cellular senescence phenotypes in response to inducing stimuli, possibly in part through SNUPN and ATXN1 degradation (Yoon et al., 2013). Both HOTAIR and p21 are downregulated by binding of the RBP HuR, which normally stabilizes mRNAs, but in the case of these lncRNAs, stimulates assembly and recruitment of a miRNA silencing complex (Let-7/Ago2) to elicit their decay (Yoon et al., 2012). Thus, cytoplasmic lncRNAs can regulate translation and protein turnover events and can be subject to miRNA-mediated regulation of their stability.

Conversely, a well appreciated mode of lncRNA function in the cytoplasm is to function as decoys for miRNAs (aka sponges; Figure 3). First described in plants where the lncRNA IPS1 sequesters miR-399 as part of the regulation of phosphate homeostasis (Franco-Zorrilla et al., 2007), several lncRNA miRNA decoys are now known, some of which harbor multiple sites for distinct miRNAs and can exist as splicing-intermediate derived circular lncRNAs (Hansen et al., 2013; Memczak et al., 2013). Many miRNA decoys appear highly regulated, with distinct tissue, developmental and environmentally responsive expression patterns, and have the potential to exhibit large-scale effects on mRNA translation and decay, owing to the diversity of miRNA molecules whose function they may impede. We guide readers to other detailed reviews on these topics (Ebert and Sharp, 2010a, b; Yoon et al., 2014; Rong et al., 2017).

### mRNAs Scaffold Co-translational Assembly of Protein Complexes

Increasing evidence indicates that mRNAs serve as scaffolding platforms for co-translational protein complex assembly (Natan et al., 2017). In prokaryotes, subunits of a given protein complex are often encoded in operons (a cluster of genes of common function under a single promoter), which are transcribed into polycistronic mRNAs. Translation of polycistronic mRNAs in turn enhances the likelihood of the encoded protein complex subunits interacting. Such interactions can occur on polycistronic mRNAs even when nascently synthesized proteins are still associated with ribosomes, owing in part to their close spatial proximity upon synthesis (Shieh et al., 2015). In this way, the mRNA serves as a scaffolding molecule facilitating nascent protein interactions. Furthermore, adjacent genes in operons tend to exhibit larger protein interaction interfaces (Koonin and Mushegian, 1996; Dandekar et al., 1998; Shieh et al., 2015; Wells et al., 2016). These findings, combined with additional bioinformatic and structural analyses of protein complex assembly suggest that operon gene order often matches (and thus may help direct) the order in which protein subunits assemble (Wells et al., 2016). Besides spatial proximity of subunits, lower stoichiometric variation in protein complex subunit expression is an additional advantage of protein complex assembly on polycistronic mRNAs (Swain, 2004; Sneppen et al., 2010; Shieh et al., 2015).

Eukaryotes lack operons, meaning any co-translational interactions must involve an *in trans* event such as recruitment of an interacting protein to a translating mRNP, or juxtaposition of translating mRNPs. Nonetheless, co-translational assembly of protein complexes in eukaryotes has been reported (Natan et al., 2017). Notably, recent affinity purification studies of well characterized protein complexes in yeast (Shiber et al., 2018) and human cells (Kamenova et al., 2019) demonstrate in many cases that nascently translating proteins interact strongly with proteins with which they are destined to form complexes with. Co-translation interactions were identified via careful interaction domain mapping, mRNA-protein co-localization microscopy and critically by detection of both protein interactors, and their mRNA template molecules, following purification of a protein complex subunit of interest. Combining nascent peptide purification with ribosome profiling (an RNA sequencing based method to map ribosome mRNA footprints) allowed determination of precisely where ribosomes were located on a given mRNA when their nascent peptides interacted with a protein of interest (Shiber et al., 2018). Co-translational protein interactions could be unidirectional (meaning Protein A and its mRNA are purified by Protein B isolation, but not vice versa) or bidirectional. This depended largely on how close a given protein’s interaction domain was to the C-terminus, as this affects the degree and timing of exposure of the interaction domain from within the translating ribosome (Shiber et al., 2018). Co-translational assembly of protein complexes was important in preventing protein misfolding and increasing the efficiency of protein complex assembly (Shiber et al., 2018; Kamenova et al., 2019). Thus, co-translational assembly of proteins in eukaryotes may be a common phenomenon.

Two primary models for eukaryotic co-translational protein interaction are generally postulated. The first states that a fully synthesized protein interacts with a nascently synthesizing protein, though whether this simply involves random diffusion, co-localization or an active chaperone driven recruiting process is unclear. A second model is that, particularly in the case of bidirectional co-translational protein interactions, translating mRNPs may be tethered to one another *in trans* by their nascent peptides (Natan et al., 2017; Schwarz and Beck, 2019). Though the above discussed examples do not distinguish between these models, recent data, discussed below, adds weight and new insight to both ideas and focuses attention on the importance of mRNA as a scaffolding molecule.

### mRNA 3′UTR Scaffolding of Protein Complexes

mRNAs possess three primary domains: a 5′ UTR, an open reading frame and a 3′UTR. While all are implicated in translation, ribosomes ordinarily only transit through 5′UTRs and ORFs, leaving 3′UTRs as regions where more stable interactions with proteins and other RNA molecules can be sustained. 3′UTR sequences are unconstrained by selection for a given protein sequence, are significantly longer than 5′UTRs, and increase in length with organismal complexity (Pesole, 2002; Mayr, 2017). Thus, it is not surprising that while 3′UTR localized interactions clearly govern individual mRNA functions, such as translation, decay and localization (Wilkie et al., 2003; Andreassi and Riccio, 2009), new functions for 3′UTRs are being uncovered.

**FIGURE 4:**
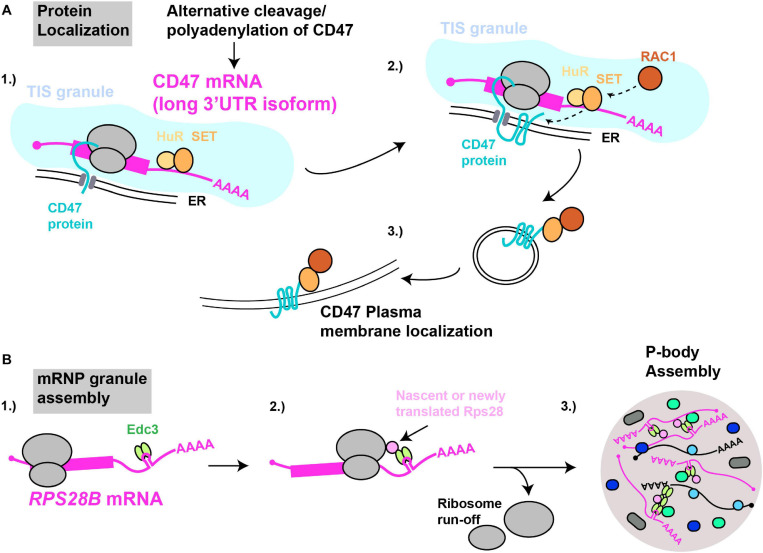
mRNAs as cytoplasmic RNA scaffolds. **(A)** Protein localization: (1) The *CD47-LU* mRNA 3′UTR recruits HuR and SET. (2) Within TIS11B-ER membrane compartments (TIS granules), nascently translated CD47’s interaction with SET is facilitated. (3) Recruitment of RAC1 by SET results in subsequent translocation of CD47 to the plasma membrane. **(B)** mRNP granule assembly: (1) The *RPS28B* 3′UTR is presumed to recruit Edc3 prior to its subsequent interaction, (2) with either nascently or newly translated Rps28. (3) Since translating mRNAs are excluded from mRNP granules, ribosome run-off is likely required for an *RPS28B*-Edc3-Rps28 RNP complex to help nucleate yeast P-body assembly.

Pioneering work by the Mayr lab demonstrated that specific mRNA 3′UTRs can scaffold protein-protein interactions in which a 3′UTR bound protein (or proteins) interacts with nascently synthesized protein translated on the mRNA itself, with important physiological consequences (Figure 4). The first report of this phenomenon (Berkovits and Mayr, 2015) concerned the CD47 gene, which encodes a protein capable of both ER and cell surface localization; at this latter site, CD47 protects cells from phagocytosis by macrophages (Oldenborg et al., 2000; Jaiswal et al., 2009). Alternative cleavage and polyadenylation usage, a highly regulated means by which alternate 3′UTR isoforms are generated (Sandberg et al., 2008; Mayr and Bartel, 2009; Lianoglou et al., 2013), results in two primary CD47 3′UTR mRNA isoforms - a short (CD47-SU) and long (CD47-LU) isoform. Knockdown of CD47-LU inhibits CD47 protein cell surface localization, independently of any mRNA localization effects, as both short and long CD47 mRNA reporters localized to the ER. Subsequent analysis of 3′UTR binding sites, gene knockdown, RNA-immunoprecipitation and mRNA-protein tethering approaches determined that HuR likely binds the CD47-LU 3′UTR in association with SET. In turn, SET interacts with nascently translated CD47 protein, and the membrane-localizing small GTPase RAC1, which then aids translocation of CD47 (and SET) to the plasma membrane (Ten Klooster et al., 2007). Underlying the importance of this 3′UTR scaffolding of a protein-localizing complex, cells forced to express either the CD47-LU or CD47-SU in isolation showed significant differences in phagocytic susceptibility (CD47-LU cells more resistant) and induction of apoptosis following γ-irradiation (only CD47-SU cells induce apoptosis) (Berkovits and Mayr, 2015).

3′UTR driven co-translational assembly of protein complexes likely occurs in all eukaryotes. We recently demonstrated in yeast that a specific mRNA 3′UTR acts as an mRNA scaffold promoting the assembly of P-bodies (Fernandes and Buchan, 2020), which are cytoplasmic membrane-less organelles enriched in non-translating mRNPs. The mRNA in question is *RPS28B*, one of two *RPS28* paralogs (which code for ribosomal protein S28) that has an unusually long 3′UTR (643 nts) and which harbors a stem-loop structural element which binds Edc3 (He et al., 2014), a protein involved in P-body assembly (Decker et al., 2007). Using genetics, microscopy, and protein interaction assays, we demonstrated that Edc3 recruited by the *RPS28B* 3′UTR binds nascently or newly translated Rps28b in a manner dependent on the *RPS28* ORF and 3′UTR being in *cis* (Figure 4). The resulting Edc3-Rps28 interaction in turn is key to normal P-body assembly.

To our knowledge, *RPS28B* is the first case of a specific mRNA implicated in scaffolding an mRNP granule, and it is likely that other RNA molecules (like NEAT1 in paraspeckles) will possess potent scaffolding potential for RNP granules, based on their potentially high interaction valency (either with proteins or other RNA molecules (Van Treeck and Parker, 2018; Van Treeck et al., 2018)) and ability to exist in a non-translating state. Indeed, recent data argues that poorly translated, long RNAs preferentially accumulate within RNP granules such as P-bodies and stress granules (Khong et al., 2017). *RPS28B* also meets these criteria (Ingolia, 2009), though questions remain as to the nature of the Rps28-Edc3 protein interaction, and whether regulation of *RPS28B* translation rates or abundance may also occur to influence P-body assembly. Finally, our work demonstrates that mRNAs from gene paralogs, in addition to transcript isoforms from a single gene, represents another means to allow mRNA-based regulation of macromolecular complex assembly. Given that 50–80% of all human genes generate mRNAs with alternative 3′UTRs (Lianoglou et al., 2013) a broad role for 3′UTRs in scaffolding co-translational protein complex assembly seems feasible (Mayr, 2019).

### mRNA Co-localization Can Aid Co-translational Protein Interactions

While mRNA localization clearly facilitates protein localization (Holt and Bullock, 2009), co-localization of mRNAs in specific cellular compartments may also aid co-translational protein interactions and/or protein complex assembly. Such a mechanism is an appealing explanation for co-translation protein assembly mechanisms discussed above. Interestingly, a few studies suggest the existence of “translation factories,” where functionally related mRNAs are spatially concentrated in cellular compartments.

In human cells, several membrane protein-encoding mRNAs, including CD47-LU (but not CD47-SU), harbor binding sites for an RBP called TIS11B. These mRNAs, as well as SET and HuR, coalesce with TIS11B in an ER-intertwined reticular structure termed TIS granules, that rely on TIS11B expression for their assembly (Ma and Mayr, 2018). Importantly, TIS11 expression, and formation of TIS granules promotes interaction of SET with CD47, and localization of CD47 to the plasma membrane. This may reflect increased retention of CD47 mRNA and SET within the TIS granule based on fluorescence recovery after photobleaching data, thus increasing the likelihood of co-translational CD47 interaction with SET (Ma and Mayr, 2018).

In yeast, specific glycolytic mRNAs (Lui et al., 2014) and select translation factor mRNAs (Pizzinga et al., 2019) co-localize in distinct translationally active granules, at least some of which encode proteins that collectively form multi-subunit complexes. Finally, several mRNAs encoding components of the Arp2/Arp3 complex, an actin cytoskeleton-regulating complex, co-localize and at least in some cases are translated at the leading edge of fibroblasts (Mingle et al., 2005; Willett et al., 2013). However, direct testing of whether mRNA co-localization effectively serves as a eukaryotic analog of a prokaryotic operon-based protein complex assembly mechanism remains a question for future study.

## Identifying New RNA Scaffolds and Decoys

Estimates in the human genome suggest between 20 and 60k loci in the human genome transcribe lncRNA (Iyer et al., 2015; Hon et al., 2017), with over 170k distinct lncRNA transcripts now reported (Zhao et al., 2021). A considerable challenge in the field lies in discriminating transcriptional noise from functional lncRNA molecules that may act as scaffolds or decoys. However, several methodologies now exist that promise more widespread detection of functional RNA scaffold and decoy molecules, including those that may exhibit conservation.

Assessing reproducibility and condition-specific changes in expression levels, as well as conservation of RNA sequence, exon number, genomic position and transcriptional level is a generally utilized approach for identifying putative functional lncRNAs, for which various amenable bioinformatic tools are now available (Derrien et al., 2012; Ulitsky, 2016; Nelson et al., 2017). LncRNAs typically exhibit a lower degree of sequence conservation and expression level than seen for most protein coding genes, making comparative genomic studies alone more challenging, though the presence of certain sequence features (transposable element-derived sequences (Wang et al., 2017), miRNA binding sites (Furió-Tarí et al., 2016)) can help predict potential scaffolding or decoy potential.

Known scaffold or decoy RNAs often harbor structured domains and typically interact with proteins. RNA-seq technologies that assess RNA secondary structure (e.g., SHAPE (Lucks et al., 2011), Structure-Seq/DMS-Seq (Ding et al., 2014)), structured RNA-regions bound by proteins (e.g., CLASH (Kudla et al., 2011)) or both (e.g., PIP-SEQ) (Foley and Gregory, 2016) may therefore provide suggestive data on a broad transcriptome-wide scale. Comparative analyses of RNA secondary or tertiary structures obtained either via structural means (e.g., NMR, X-ray crystallography), RNA-seq methods or solely via bioinformatic sequence prediction can also help identify functional RNA scaffolds or decoys. While structural methods are clearly limited in throughput, they represent the gold standard in reliably defining the presence of an RNA structural element and identifying sequence targets for subsequent genetic perturbation and functional analysis.

Genetic screening remains a powerful initial tool to identify functional lncRNAs with possible scaffolding or decoy functions. Genome wide studies directly targeting lncRNA loci via CRISPR-Cas9 means (Joung et al., 2017; Liu et al., 2017), and a study of 285 lncRNAs degraded in the nucleus by Antisense oligonucleotide (ASO) mediated RNase-H degradation (Ramilowski et al., 2020) have revealed growth phenotypes in about 0.1–7% of cases. Cell-line and context specific phenotypes may explain this relatively low percentage (Joung et al., 2017; Liu et al., 2017). Indeed a larger fraction of ASO-depleted lncRNAs revealed significant alterations in transcriptome composition, often in the absence of growth phenotypes (Ramilowski et al., 2020). Though not commonly utilized, discrimination of nuclear versus cytoplasmic lncRNA functions may also be achieved in principle by siRNA knockdown, which only targets cytoplasmic RNA species for degradation.

Another means of identifying RNA scaffolds or decoys is to biochemically isolate their interacting biomolecular partners and co-purify and sequence the RNA. For example, isolation of the telomerase complex (via chromatography, TERT enzyme pulldown) has been used to identify or validate prediction of TR RNAs in several species (Greider and Blackburn, 1989; Feng et al., 1995; Dew-Budd et al., 2020). Cross-linking and Immunoprecipitation methods (Lee and Ule, 2018) can also be applied not only to detect putative interacting RNA scaffolds or decoys, but also the specific location on the RNA where the protein binds. This in turn may help identify key RNA sequence/structural elements to base comparative genomic analyses upon when determining if an RNA scaffold or decoy may be functionally conserved.

Finally, microscopy-based analyses using FISH, in combination with immunohistochemistry of other cellular organelles or proteins, can provide insight as to functional and regulatory mechanisms of lncRNAs. A study of 61 lncRNAs in various human cell types revealed that while most were nuclear, highly diverse patterns of localization were observed (Cabili et al., 2015). Notably, nuclear lncRNA foci dissolve during mitosis and become generally dispersed; what the functional impacts of this dispersal process are, and how nuclear localization is re-established remain unclear.

The above methods can also be applied to identifying mRNA scaffold and decoy molecules, though separating 3′UTR-based regulation of nascent protein-interaction from 3′UTR based regulation of the mRNA (i.e., mRNA translation, stability, localization) provides significant additional challenges. Thus, perturbation or forced expression of a mRNA 3′UTR isoform of interest, coupled to assessment of protein interactions (e.g., Co-immunoprecipitation, unbiased mass spectrometry) should include control experiments to verify no alteration of mRNA function or overall protein expression.

It is clear there is no one single path to identification of functional RNA scaffolds and decoys. While sequencing, bioinformatic and comparative analyses studies generally offer the most rapid means to identify putative RNA scaffolds and decoys, genetic, biochemical and microscopy methods can all be brought to bear to enhance mechanistic understanding.

## Conclusion and Future Directions

The evidence is mounting that RNA molecules ubiquitously function as scaffolds and decoys bringing together or sequestering specific biomolecules to regulate macromolecular complex assembly. LncRNAs primarily accumulate in the nucleus (Derrien et al., 2012; Cabili et al., 2015), which has led to a historical focus on their roles as RNA scaffolds and decoys in this compartment. However, lncRNAs also accumulate, and in some cases are preferentially enriched, in the cytoplasm (Gudenas and Wang, 2018). This, together with emerging data that cytoplasmic mRNAs can also play scaffolding roles suggests that many new cytoplasmic scaffolding and decoy functions for RNA molecules await discovery, aided by the paradigms provided by their nuclear counterparts.

RNA scaffolds and decoys enable dynamic and condition-specific responses to a variety of cellular stimuli (Table 1), with lncRNAs notably exhibiting significant developmental and tissue-specific control of expression (Derrien et al., 2012; Sarropoulos et al., 2019). However, detailed understanding of how synthesis, decay, and localization (when acting *in trans*) of RNA scaffolds and decoys is regulated in response to changing cellular environments is lacking in most cases. Additionally, while several RNA scaffolds and decoys, particularly within the category of lncRNAs, have been identified and functionally characterized in mammalian model systems, fewer characterized examples exist in other model systems. How, or indeed if, RNA-scaffolded complexes are actively assembled and disassembled is also unclear, though we speculate that RNA helicases may play a prominent role given their RNP-remodeling roles in other RNA biology processes and at least one prior example of precedent (Wongtrakoongate et al., 2015).

A unique feature of mRNAs is their potential to scaffold nascent protein interactions by virtue of the fact they also serve as protein synthesis templates. To date, characterized cytoplasmic mRNA scaffolds are driven by the presence of unique 3′UTR sequences generated by alternative cleavage and polyadenylation or paralogous gene transcription. Distinct mRNA 3′UTR sequences, with binding sites for nascent protein interaction partners can then help define a nascent protein’s interaction fate and function. How widespread this mode of facilitating protein-protein interaction is remains unknown, though it offers several potential advantages. For instance, protein interaction partners and their respective template mRNAs do not have to be as carefully orchestrated for simultaneously translation and co-localization to avoid competing off-target interactions. A nascent protein’s interaction fate could also be pre-determined by loading of its mRNA’s 3′UTR, possibly in the nucleus, with suitable nascent protein interaction partners. Additionally, this approach may facilitate generation of several distinct nascent protein subcomplexes, as occurs with BIRC3 (Burger et al., 2005; Holt and Bullock, 2009). A caveat of this mechanism might be a limited capacity of any 3′UTR to store (or reacquire) a supply of nascent protein interactors. Thus, this mechanism may favor assembly of low-frequency, regulatory protein interactions, or be more prevalent on poorly translated mRNAs.

Identifying new RNA scaffolds and decoys remains a significant challenge. Nonetheless, new advances in sequencing, bioinformatics and protein-RNA interaction methods promise progress toward this goal. Given the intrinsic advantages of RNA as a scaffolding molecule, and that many RNA scaffolds incorporate other functions (e.g., catalysis, modifying protein activities, guide and template functions; Table 1), we anticipate that intriguing and impactful new modes of RNA-scaffolded interactions await discovery.

## Author Contributions

NF and JRB conceived and wrote the review and generated the figures and table. Both authors contributed to the article and approved the submitted version.

## Conflict of Interest

The authors declare that the research was conducted in the absence of any commercial or financial relationships that could be construed as a potential conflict of interest.
